# Supplementary material for: Association between foot thermal responses and shear forces during turning gait in young adults
Source: PeerJ. 2021 Jan 18;9:e10515. doi: 10.7717/peerj.10515 (PMC7819117; doi:10.7717/peerj.10515)
Supplement: Supplemental Information 2 — A checklist was developed to determine external influences on our region of interest, developed by Moreira et al.,. The items in the checklist were assessed within our experiment procedures. [file peerj-09-10515-s002.pdf]

Figure S1. Thermal Imaging Checklist (Adapted from Moreira et al., 2017).

1. “The relevant individuals data of the participants must be provided. “

Relevant information (e.g., age, sex, body mass, height, body mass index and activity profile) was reported. However, we did not report the ethnicity of individuals nor whether individuals were smokers or not.

2. “Participants should be instructed to avoid alcoholic beverages, smoking, caffeine, large meals, ointments, cosmetics and showering for four hours before assessment. Also sunbathing should be avoided before assessment.”

Participants were not given specific instructions before the assessment.

3. “Extrinsic factors affecting skin temperature should be clearly described.”

Extrinsic factors (e.g., physical activity prior to the assessment, massage, electrotherapy, ultrasound, cold or heat therapy, or cryotherapy) potentially affecting skin temperature were not recorded.

4. “Ambient temperature and relative humidity of the location where the assessment took place must be recorded and reported as mean ± standard deviation.”

Ambient temperature and relative humidity of the location were reported.

5. “The assessment should be completed away from any source of infrared radiation or airflow.”

No sources of radiation or airflow were not expected. Sources of radiation or airflow were not reported.

6. “The manufacturer, model and accuracy of the camera used should be provided.”

The manufacturer, model and accuracy of the camera were reported.

7. “Any acclimation period in the examination room should be completed.”

An acclimatization period was reported.

8. “If necessary the camera should be turned on for some time prior to the test to allow sensor stabilization following the manufacturer’s guidelines.”

The necessary time for the camera to be turned on for a minimum time was reported.

9. “Conditions of image recording such as mean distance between object and camera, percentage of region of interest within the image should be detailed.”

Mean distance between object and camera were reported. Percentage of region of interest was not reported.

10. “The camera should be positioned perpendicular to the object of interest.”

The camera was positioned perpendicular to the object of interest and was reported.

11. “Emissivity settings of the camera must be provided.”

Emissivity setting of the camera was reported.

12. “The time of the day at which the images were taken should be reported.”

The time in which images were collected were not reported.

13. “The standard body position of the subject and the regions of interest must be well described and appropriately selected. A visual example is recommended.”

The standard body position and regions of interest were reported.

14. “If the skin is dried, the drying method should be clearly described.”

Unclear.

15. “The evaluation of thermograms and collection of temperature from the software should be clearly described.”

The evaluation of the thermal images and collection of temperature from the software were reported.

References

Moreira, D. G., Costello, J. T., Brito, C. J., Adamczyk, J. G., Ammer, K., Bach, A., Costa, C., Eglin, C., Fernandes, A. A., Fernández-Cuevas, I., Ferreira, J., Formenti, D., Fournet, D., Havenith, G., Howell, K., Jung, A., Kenny, G. P., Kolosovas-Machuca, E. S., Maley, M. J., Merla, A., … Sillero-Quintana, M. (2017). Thermographic imaging in sports and exercise medicine: A Delphi study and consensus statement on the measurement of human skin temperature. *Journal of thermal biology*, **69**, 155–162.
