# Supplementary figures and images for: Association between foot thermal responses and shear forces during turning gait in young adults
Source: PeerJ. 2021 Jan 18;9:e10515. doi: 10.7717/peerj.10515 (PMC7819117; doi:10.7717/peerj.10515)

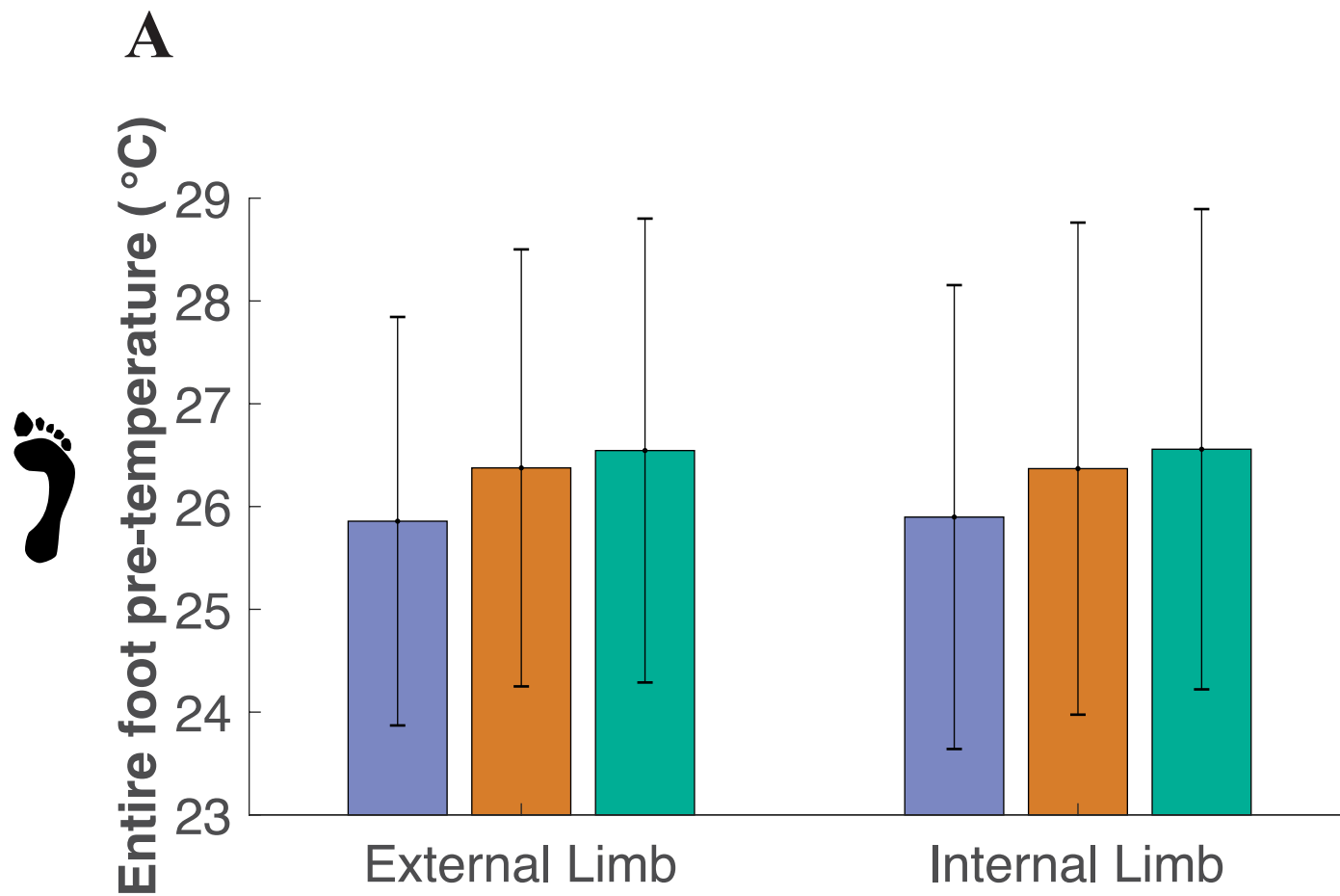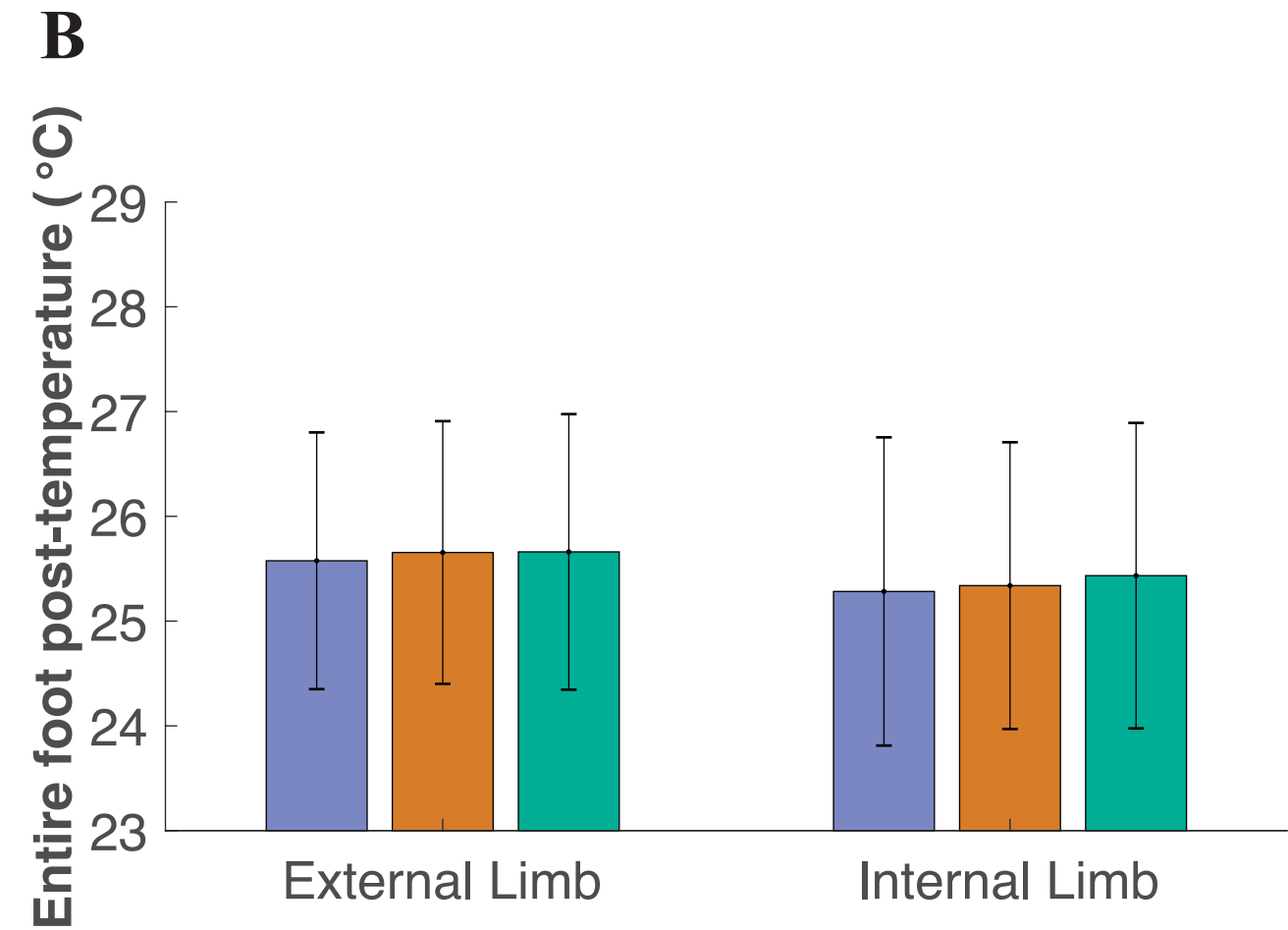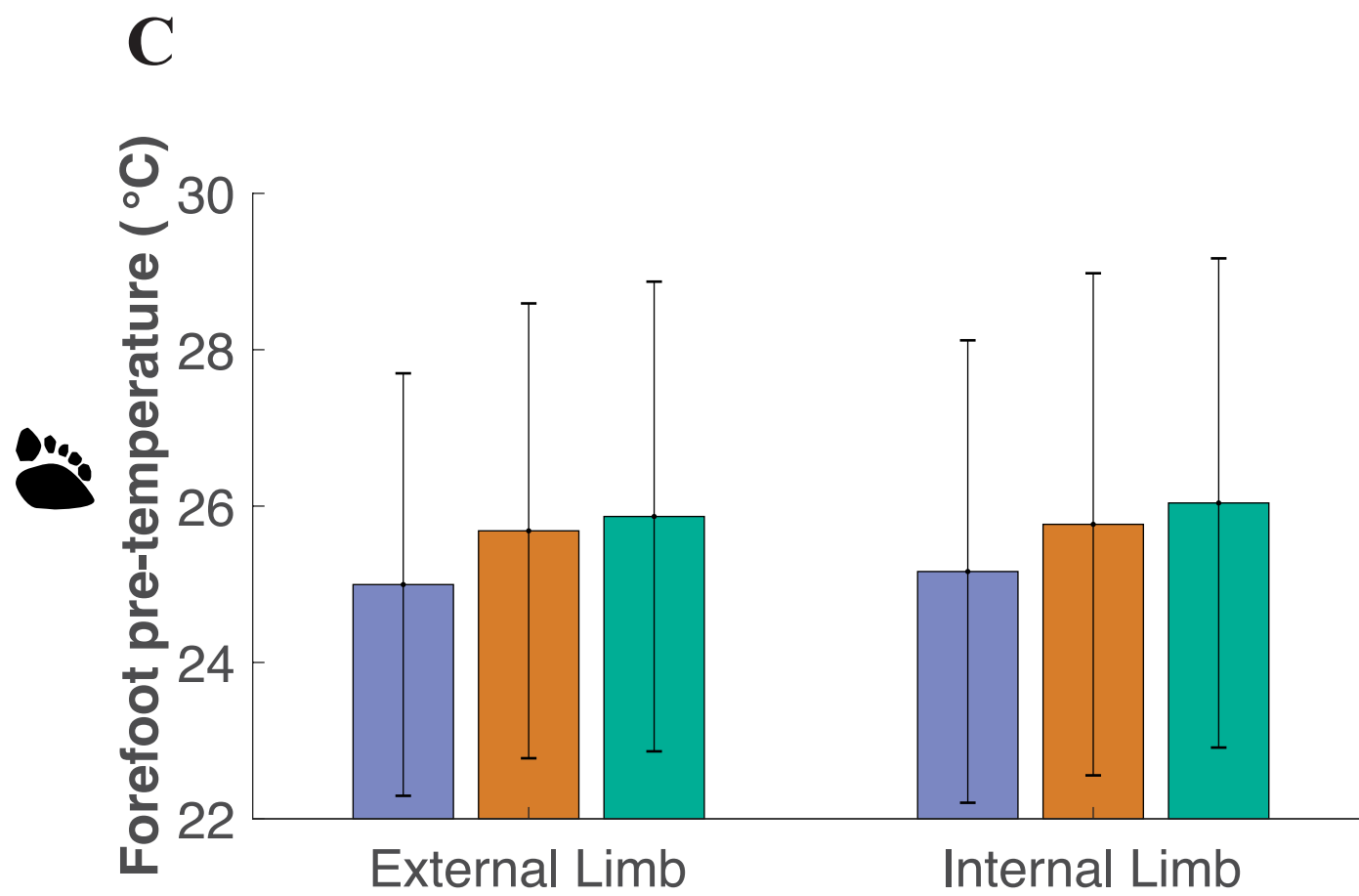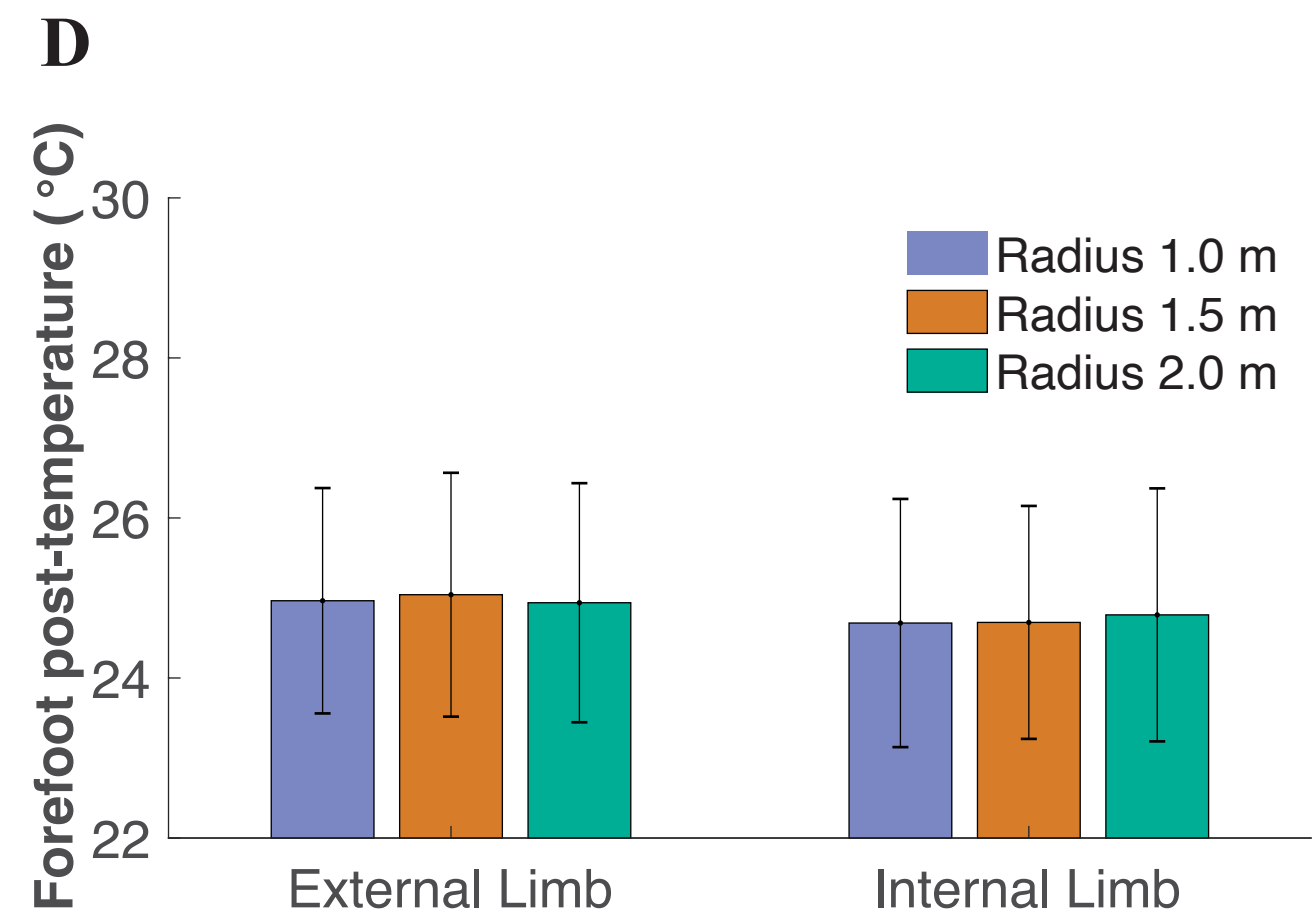

Supplement: Supplemental Information 3 — A 2 way ANOVA was utilized to determine differences in pre-temperature (baseline) between limbs and radii conditions. Baseline values increased with greater radius within the entire foot (P < 0.01) (Fig. S2 A) but not within the forefoot (P = 0.06) (Fig. S2 C). No significant differences were found between external and internal limbs in baseline temperatures within the entire foot (P = 0.90) nor forefoot (P = 0.46). No differences in post-temperature were determined between radius within the entire foot (P = 0.61) (Fig. S2C) or within the forefoot (P = 0.96) (Fig. S2D). No differences in post-temperature were determined between external and internal limbs within the entire foot (P = 0.06) nor forefoot (P = 0.10). (Values are means ± S.D.). [file peerj-09-10515-s003.pdf]

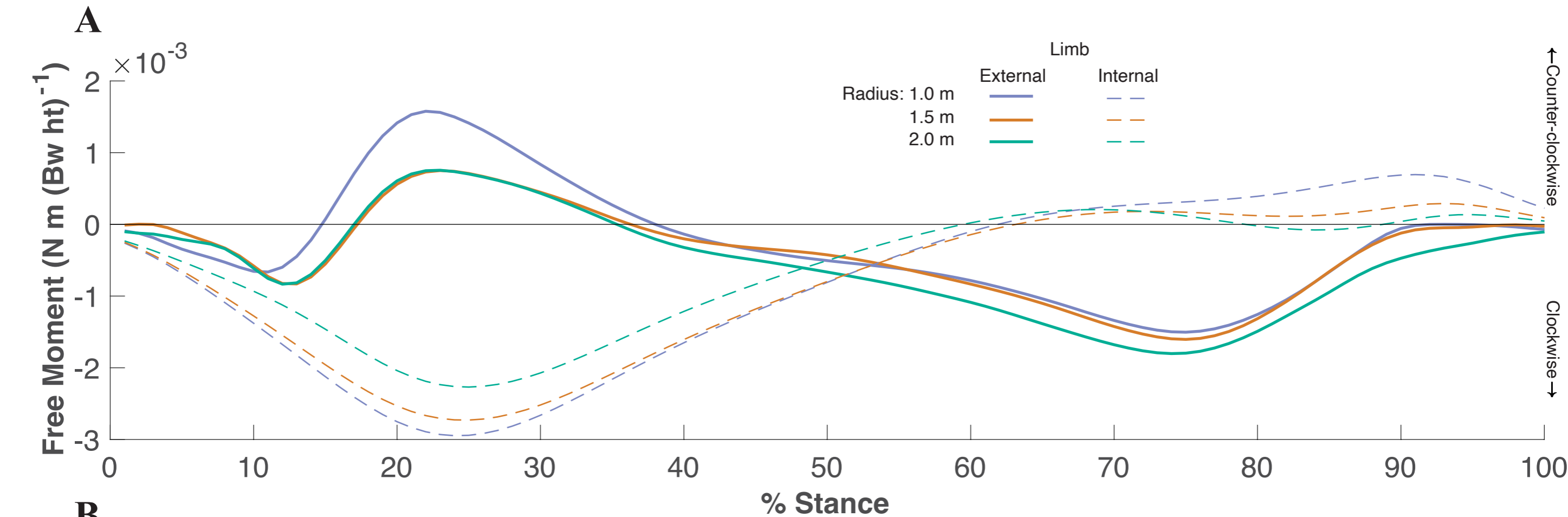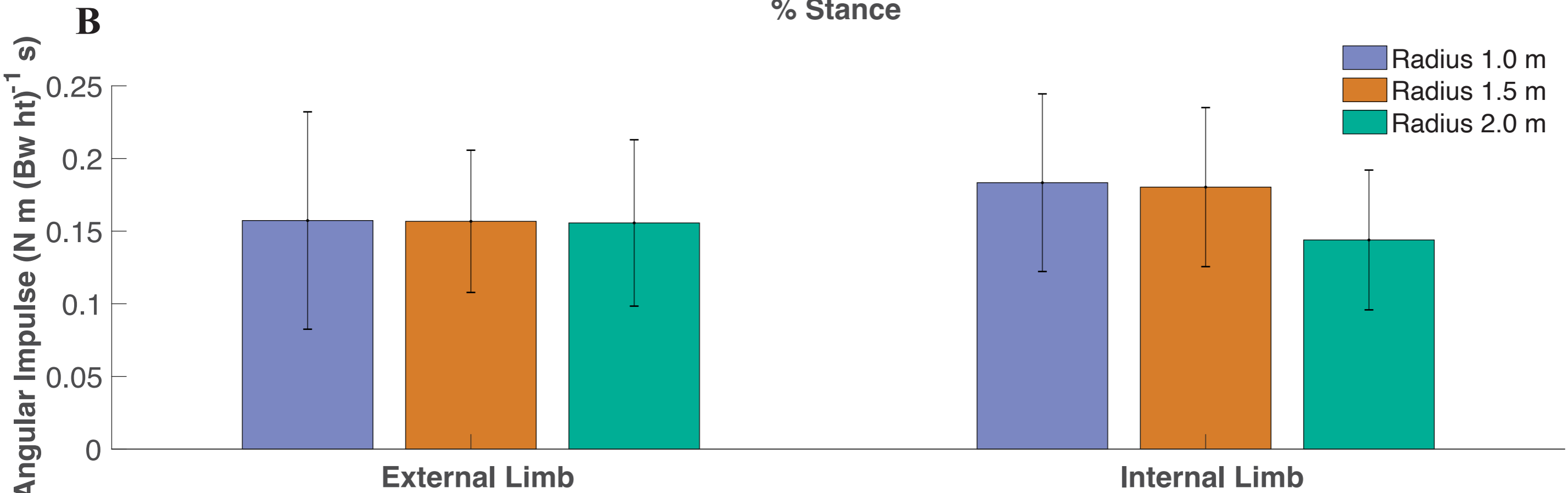

Supplement: Supplemental Information 4 — Free moment was normalized by body weight x height. Fig. S4 A demonstrates negative values when the limb has a clockwise moment (i.e., towards the turning direction) and positive values when the limb has a counter-clockwise moment (i.e., away from the direction of the turn). A 2 way ANOVA was utilized to determine differences between conditions and limbs where each were used as factors. Free moment angular impulse was not significantly different between limbs (p = 0.29) nor between radii conditions (p = 0.19). (Values are means ± S.D.). [file peerj-09-10515-s004.pdf]

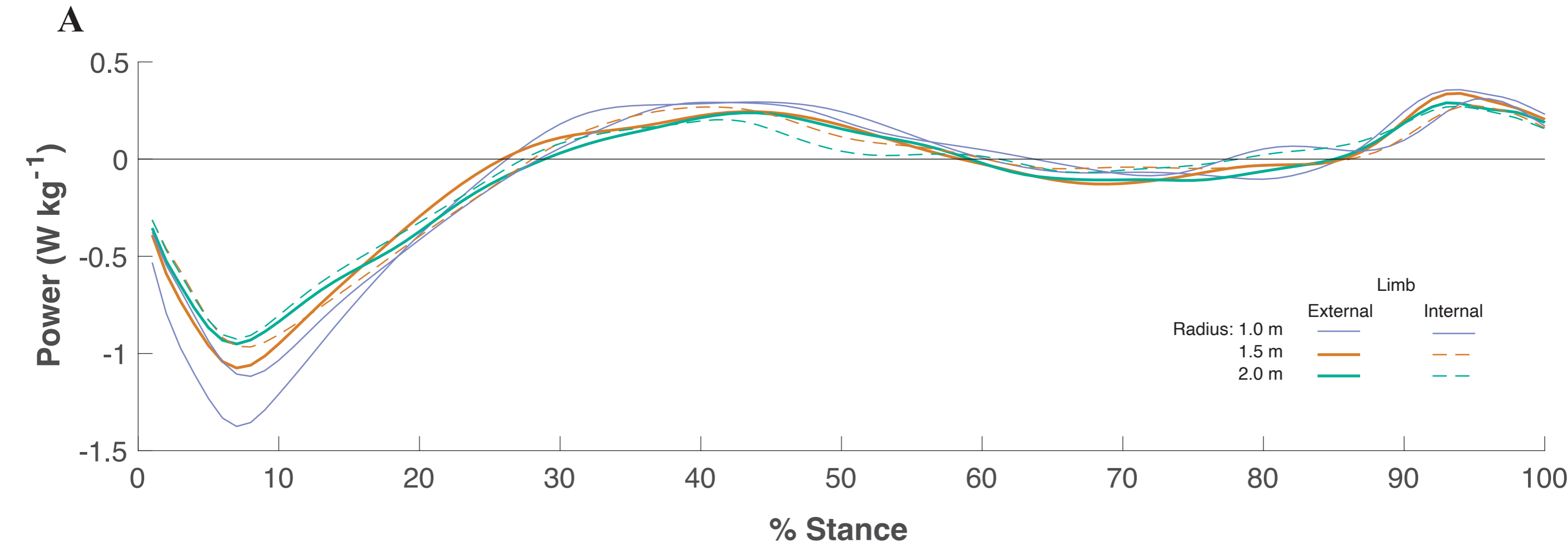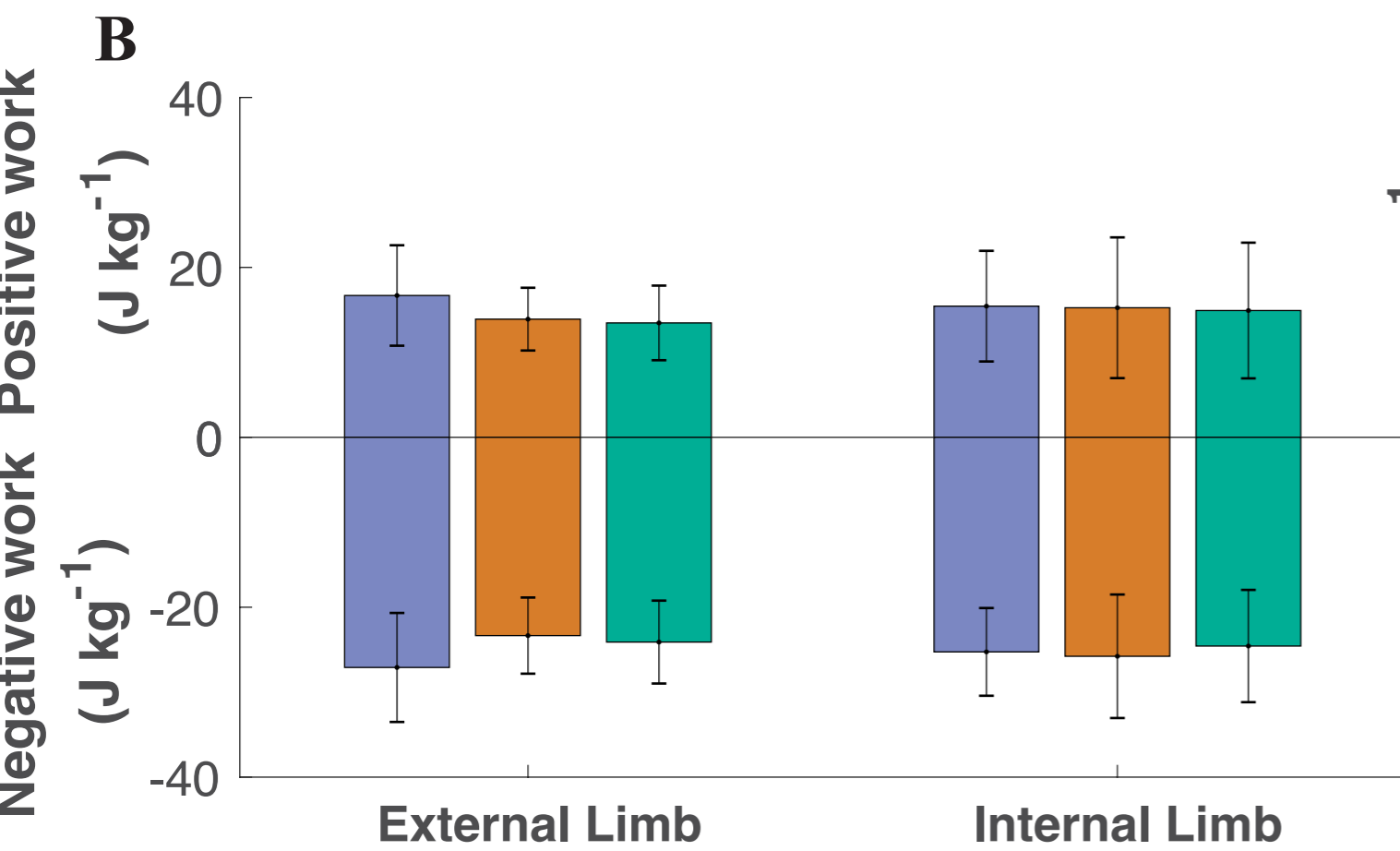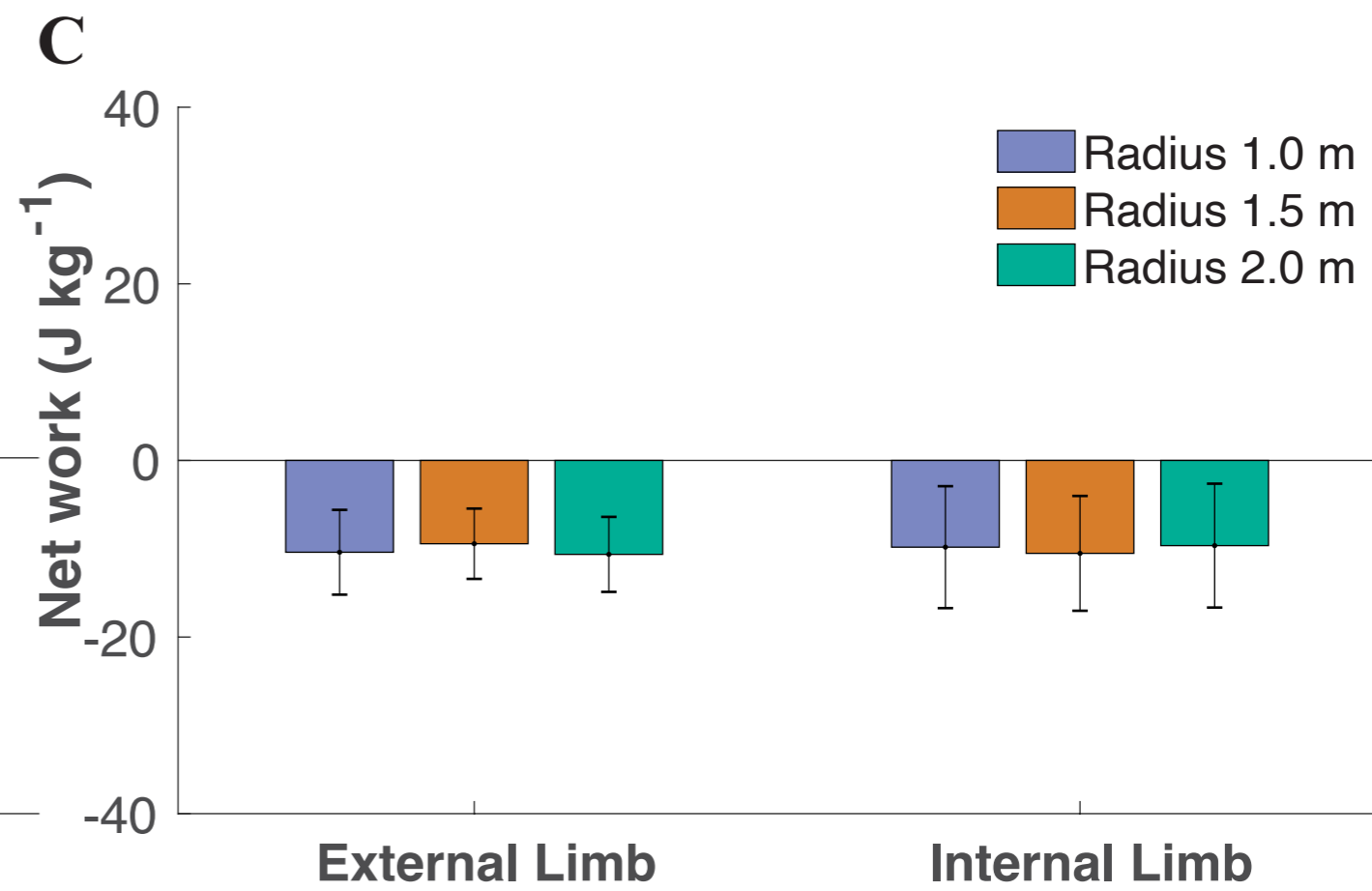

Supplement: Supplemental Information 5 — Power was normalized by body mass. A 2 way ANOVA was utilized to determine differences in foot net work (Fig. S2C) between conditions and limbs. Net work was not significantly different between limbs (p = 0.92) nor between radii conditions (p = 0.97). (Values are means ± S.D.). [file peerj-09-10515-s005.pdf]
